# Molecular mechanisms of cell proliferation induced by low power laser irradiation

**DOI:** 10.1186/1423-0127-16-4

**Published:** 2009-01-12

**Authors:** Xuejuan Gao, Da Xing

**Affiliations:** 1 MOE Key Laboratory of Laser Life Science & Institute of Laser Life Science, South China Normal University, Guangzhou 510631, PR China

## Abstract

Low power laser irradiation (LPLI) promotes proliferation of multiple cells, which (especially red and near infrared light) is mainly through the activation of mitochondrial respiratory chain and the initiation of cellular signaling. Recently, the signaling proteins involved in LPLI-induced proliferation merit special attention, some of which are regulated by mitochondrial signaling. Hepatocyte growth factor receptor (c-Met), a member of tyrosine protein kinase receptors (TPKR), is phosphorylated during LPLI-induced proliferation, but tumor necrosis factor alpha (TNF-alpha) receptor has not been affected. Activated TPKR could activate its downstream signaling elements, like Ras/Raf/MEK/ERK, PI3K/Akt/eIF4E, PI3K/Akt/eNOS and PLC-gamma/PKC pathways. Other two pathways, ΔΨm/ATP/cAMP/JNK/AP-1 and ROS/Src, are also involved in LPLI-induced proliferation. LPLI-induced cell cycle progression can be regulated by the activation or elevated expressions of cell cycle-specific proteins. Furthermore, LPLI induces the synthesis or release of many molecules, like growth factors, interleukins, inflammatory cytokines and others, which are related to promotive effects of LPLI.

## Background

Cell proliferation is a very important physiological effect for low power laser irradiation (LPLI) used in clinical practice. Increased proliferation after LPLI has been shown in many cell types in vitro, including fibroblasts from different systems [1-8], keratinocytes [9], human osteoblasts [10], calvaria osteoblast-like cells [11], lymphocytes [12], mesenchymal stem cells (MSCs) and cardiac stem cells (CSCs) [13], rat Schwann cells [14], aortic smooth muscle cell (SMC) [15], endothelial cells from veins [16] and arteries [17,18], quiescent satellite cells[19,20], human lung adenocarcinoma cells (ASTC-a-1) [21] and HeLa cells [22]. However, the mechanisms of cell proliferation induced by LPLI are poorly understood.

Various mechanisms for the mitogenic effects of low power laser irradiation have been proposed, including ligand-free dimerization and activation of specific receptors that are in the "right energetic state" to accept the laser energy, leading to their autophosphorylation and downstream effects [23], activation of calcium channels resulting in increased intracellular calcium concentration and cell proliferation [24-30]. Red to near infrared light is thought to be absorbed by mitochondrial respiratory chain components, resulting in the increase of reactive oxygen species (ROS), and adenosine triphosphate (ATP)/or cyclic AMP, and initiating a signaling cascade which promotes cellular proliferation and cytoprotection [9,12,23,24,31-37]. Following increased ATP and protein synthesis after LPLI, the expressions of growth factors and cytokines increase and ultimately lead to cell proliferation [38,39]. Studies have also shown that light irradiation can alter cellular homeostasis parameters, such as pHi, the redox state of the cell, and expression of redox-sensitive factors like NF-κB, which can lead to a proliferation increase [23,40-42].

Recently, a large number of signaling proteins reported play an important key role in the process of LPLI-induced cell proliferation, probably due to the fact that the molecular events they are involved in are the basic response of the cells to extracellular stimuli. Therefore, we believe that the investigation of the molecular events induced by LPLI could eventually reveal the mechanisms of LPLI. This is the possible reason why more and more researchers are devoted to this subject. In this paper, we firstly discuss the discovered mitochondrial photoacceptors and nonmitochondrial photoacceptors, respectively; then we review the studies on the molecular mechanisms of LPLI-induced proliferation since January 1999, which will serve as a reference for the researchers in this field.

## Review

### Photoacceptor for low power laser

#### Mitochondrial photoacceptors

Photosensitivity might be a common property of higher animals and could have physiological significance [43]. The light must be absorbed by the endogenous chromophores of cells or tissues for actions. Mitochondria are the center of many diverse cellular functions integrating signals between the organelle and the nucleus. It was suggested as early as 1981 that photosensitivity might be a common mitochondrial property in higher animals [42]. Irradiation of red and near infrared light can lead to the activation of mitochondrial respiratory chain components and the initiation of a signaling cascade which promotes cellular proliferation and cytoprotection [9,23,32]. Photon absorption causes a shift in the molecular configuration of the photoacceptor, accompanying with an associated alteration in the molecular signal of the cell [44]. The alterations in photoacceptor function are the primary reactions, and the subsequent alterations in cellular signaling and cellular functions are secondary reactions [45]. The primary reactions after light absorption are reviewed previously [23,44]: including singlet-oxygen hypothesis, redox properties alteration hypothesis, nitric oxide hypothesis, transient local heating hypothesis and superoxide anion hypothesis. The secondary reactions after light absorption are cellular signaling pathways, mitochondrial retrograde signaling included [42]. The mitochondrial retrograde signaling is the communication in cells from mitochondria to the nucleus that influences many cellular activities under both normal and pathophysiologic conditions [46,47]. This recently discovered signaling pathway is opposite to a common and well-defined pathway transforming information from the nucleus and cytoplasm to the mitochondria [48]. Mitochondrial retrograde signaling is initially defined by the alteration of mitochondrial membrane potential (ΔΨm) [46]. Later, other characteristics like changes in the concentration of mitochondrial ROS and Ca^2+ ^are introduced. The modulation of elements of mitochondrial retrograde signaling (ΔΨm, ROS, Ca^2+^, NO^•^, pH_i_, fission-fusion homeostasis of mitochondria) by the irradiation are reviewed recently [42].

There is now a growing body of evidences indicate that low-intensity red and near-infrared light is acting on cells through a primary photoacceptor: cytochrome C oxidase, the terminal enzyme of the mitochondrial electron transport chain [23,43,49-53]. Absorption of light by cytochrome c oxidase can increase ΔΨm, ATP and ROS, leading to increased energy availability and signal transduction [39,43,54,55]. These biochemical and cellular changes lead to macroscopic effects, such as increased cell proliferation or accelerated wound healing [39,51,56]. Cell proliferation of mammalian cells is widely used for exploring the changes after LPLI. The initial phase of the cell proliferation, adhesion of cells to a matrix (attachment), as well as DNA and RNA synthesis rate are the cellular responses studied most systematically [42]. Thus, coincident action spectra [23,43,50] for processes in the nucleus (DNA and RNA synthesis) and the plasma membrane (attachment) with the absorption spectra of potential photoacceptors, together other evidences [32,49,55] prove that cytochrome c oxidase is a primary photoacceptor of light in the red to near infrared region.

Cytochrome c oxidase of mammalian cells is a large multicomponent membrane protein (molecular size 200 kDa). Its possible absorbing chromophores for visible light are two heme moieties (heme a and heme a3), two redox-active copper sites (Cu_A_, and Cu_B_,), one zinc, and one magnesium [23]. It has been showed that fully oxidized or fully reduced cytochrome c oxidase cannot be considered as a primary photoacceptor but only when it is in one of the intermediate forms (partially reduced or mixed-valence enzyme) [23]. Under normal physiological conditions, fully oxidized or fully reduced cytochrome c oxidase is not present, but only more reduced or less reduced (less oxidized or more oxidized) forms [50]. In the process of respiration, the Cu_A _chromophore of cytochrome c oxidase (more exactly, a pair of Cu_A _chromophores) accepts electrons from the cytochrome c molecule; internal electron transfer among Cu_A_, heme a, and the heme a3-Cu_B _center results in reduction of molecular oxygen via at least seven redox intermediates [57]. Thus, it is unclear which one of the cytochromes c oxidase intermediates is the primary photoacceptor.

Other study shows that low-level laser therapy (AsGa, wavelength of 904 nm) of animals significantly increases the activities of complexes II and IV of mitochondrial respiratory chain components but does not affect succinate dehydrogenase (SDH, a part of complex II) activity [58]. In contrast to red and near-infrared light, in the blue spectral region, flavoproteins such as NADH-dehydrogenase, a part of complex I of mitochondrial respiratory chain components, can work as photoacceptors [59].

The prosthetic group of heme in cytochromes b, c1 and c is iron protoporphyrin IX (PpIX), the same heme as in myoglobin and hemoglobin [60], which has been reported to have high absorption of green light. Absorption of laser light energy by PpIX causes photobleaching due to its photodynamic action. In recent study, after low power green laser (532 nm, 30 mW) irradiation, ΔΨm of B-14 cells is significantly increased (by 13%). Fluorescence emission spectra analysis shows that fluorescence maximum at 635 nm, corresponding to emission of PpIX, is significantly lower in irradiated mitochondria suspensions, suggesting that PpIX is photobleached by low power green laser irradiation [61]. Thus, it is possible that PpIX of cytochromes b, c1 and c absorbs the green light.

#### Nonmitochondrial photoacceptors

Enhancement of cellular metabolism via activation of nonmitochondrial photoacceptors has been reviewed in detail elsewhere [44]. Briefly, in phagocytic cells irradiation initiates a nonmitochondrial respiratory burst (production of ROS, especially superoxide anion) through activation of NADPH-oxidase located in the plasma membrane [44]. Another important redox chains are NO-synthases, a group of redox-active P450-like flavocytochromes that are responsible for generation of NO under physiological conditions [62]. But, the irradiation effects on these systems have not been investigated well.

Recent study shows that in cardiac and sperm cells after the halogen lamp irradiation, only the 400–500 nm range of visible light can produce oxyradicals. The endogenous photosensitizer is found predominantly in the cytoplasm and is smaller than 12 kD [63]. Flavins, a small and water soluble photosensitizer active only at wavelengths shorter than 500 nm, have previously been found to be responsible for visible light induction of free radical reactions in cell medium [64]. The researchers suggest that flavins are responsible for the photosensitization of the observed oxyradicals in cells [63]. Flavin is present in cells primarily as flavin adenine dinucleotide (FAD) and flavin mononucleotide (FMN). Most flavin molecules in the cell are bound to proteins, and this may reduce their reactivity [63].

The photobiological action is mainly through the activation of the respiratory chain of mitochondria, thus, we focus mainly on the components of mitochondrial respiratory chain. Reported primary photoacceptors include cytochromes c oxidase (red and near-infrared light region), NADH-dehydrogenase (blue spectral region), and cytochromes b, c1 and c (green light region). Their cofactors are porphyrins (for cytochromes c oxidase and cytochromes b, c1 and c), or flavins (for NADH-dehydrogenase), respectively. Thus, it is possible that photoabsorbing molecules in the cell like porphyrins and flavoproteins (some respiratory-chain components belong to these classes of compounds) can be reversibly converted to photosensitizers [65].

How are the primary reactions of photons with photoacceptors in the respiratory chain connected with DNA and RNA synthesis in the nucleus or with changes in the plasma membrane? The principal answer is that between these events are secondary reactions (cellular signaling cascades or photosignal transduction and amplification chain) [44]. The elements of mitochondrial retrograde signaling (ΔΨm, ROS, Ca^2+^, NO^•^, pH_i_, fission-fusion homeostasis of mitochondria) are involved in these secondary cellular signaling cascades. The changes of these elements of mitochondrial retrograde signaling mediate the activation or suppression of signal molecules in the cytoplasm and subsequent changes of downstream cascades, leading to the synthesis of DNA, RNA, proteins and enzymes in the nucleus and cytoplasm or the changes in the plasma membrane. Finally, these changes result in the photobiological effects of cells like proliferation and differentiation.

### Signal transduction pathways involved in LPLI-induced cell proliferation

Now, we mainly review the involvements of signal molecules in secondary cellular signaling in respond to light irradiation. Some of these reported signal molecules are related to the changes of elements of mitochondrial retrograde signaling. Whether other reported signal molecules are related to the changes of elements of mitochondrial retrograde signaling need to be investigated further.

#### TPKR/Ras/Raf/MEK/ERK/Mnk1/eIF4E/CyclinD1 pathway

**Figure 1:**
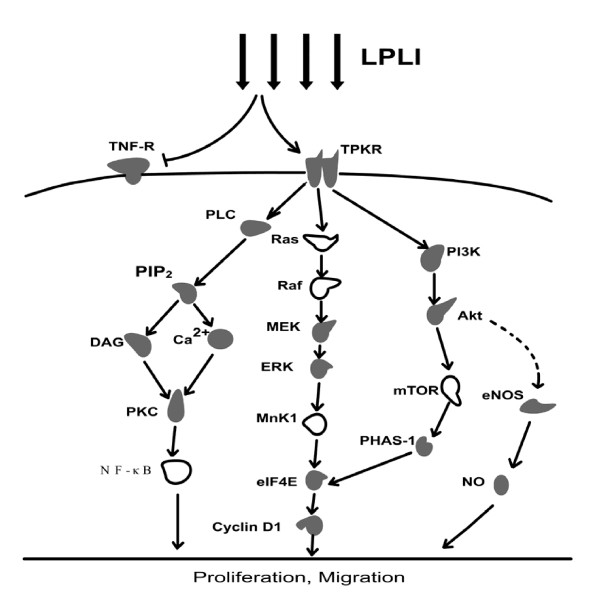
**Signal pathways involved in LPLI-induced cell proliferation**. Solid shapes mean the involvements of these molecules have been reported; Hollow shapes mean the involvements of these molecules have not been reported. Broken lines mean that the downstream changes didn't produce by the upstream directly.

In mammalian cells, there are at least three distinct MAPK cascades that are activated by different extracellular stimuli. The extracellular signal-regulated protein kinase (ERK) pathway appears to play an important role in growth-factor-induced proliferation, differentiation and cellular transformation [66]. Upon epidermal growth factor (EGF) stimulation, the sequential activation of small GTPase Ras protein, the Raf-1, the MAPK kinase (MEK1/2) and two ERK isoforms (ERK1/2) are involved [66]. Two other MAPK cascades are the stress-activated protein kinases (SAPKs)/Jun N-terminal kinases (JNKs) and p38 MAPK, which can be activated by stress signals like heat shock, UV irradiation, high osmolarity and proinflammatory cytokines (tumor necrosis factor α, TNF-α) [67,68]. It has been reported that LPLI (He-Ne laser, 632.8 nm) could induce the phosphorylation of tyrosine protein kinase receptor (TPKR) (such as c-Met, receptor of hepatocyte growth factor), previously shown to activate the MAPK/ERK pathway, and promote proliferation of the cells [69]. However, LPLI has no effects on TNF-α receptor which activates the p38 and JNK pathways [69]. Subsequently, by phospho-specific antibodies, the activation of MAPK/ERK, but not JNK and p38 MAPK kinases, are detected after LPLI stimulation [69]. Thus, through specific receptor phosphorylation, LPLI does not work via a stress signal pathway but rather a mitogen-activated pathway that leads to the activation and proliferation of quiescent satellite cells (Fig. 1).

Eukaryotic initiation factor 4E (eIF4E) is a major regulator of cap-dependent mRNA translation in response to proliferative stimuli like hormones, growth factors and mitogens [70,71]. The regulation of eIF4E protein are mainly through three mechanisms: (a) regulation of eIF4E expression [72]; (b) phosphorylation of eIF4E [73]; and (c) phosphorylation-dependent dissociation of the translational-repressor protein from eIF4E (PHAS-I: protein heat and acid stable, also as eIF4E binding protein-1 4EBP1) [74,75]. The non- or partial phosphorylation form of PHAS-I inhibit the activity of eIF4E by strongly interacting with eIF4E. LPLI stimulation increases the phosphorylation of eIF4E, but doesn't affect the expression level of eIF4E [76]. Also, LPLI elevates the phosphorylation of its inhibitory binding protein PHAS-I, as well as the expression of cyclin D1. These results suggest that the mechanisms (b) and (c) are involved in the activation of eIF4E induced by LPLI and that protein translation is initiated during the process of LPLI treatment [76]. However, in the presence of the MEK inhibitor, PD98059, LPLI-induced ERK1/2 activation and eIF4E phosphorylation are abolished and expression of cyclin D1 is dramatically reduced [69,76] (Fig. 1).

Although eIF4E phosphorylation is through Ras/Raf/ERK pathway, but eIF4E is not directly activated by MAPK/ERK. The eIF4E can be directly phosphorylated by mitogen-activated protein kinase-interacting kinase 1 and 2 (Mnk1 and Mnk2), and the latter are phosphorylated by p38 MAPK or MAPK/ERK [77-79]. The authors proposed that Mnk1 is responsible for the phosphorylation of eIF4E [76] (Fig. 1). Thus, LPLI initiates protein translation and induces cell proliferation via the TPKR/Ras/Raf/MEK/ERK/Mnk1/eIF4E/Cyclin D1 pathway.

#### TPKR/PI3K/Akt/mTOR/eIF4E pathway

Phosphoinositide 3-kinases (PI3Ks) are important downstream kinases of transmembrane TPKR. The involvement of PI3K in the LPLI-induced cell proliferation is proved by employing the inhibitor of PI3K, wortmannin [76]. Akt phosphorylation is enhanced upon LPLI (He-Ne laser, 632.8 nm) stimulation and is attenuated in the presence of wortmannin [76]. The mammalian target of rapamycin (mTOR), a downstream kinase in the PI3K pathway, is thought to phosphorylate PHAS-I [80]. The authors propose that the activated Akt directly phosphorylates mTOR, which in turn induces PHAS-I phosphorylation [76,81,82]. The phosphorylated PHAS-I dissociates from eIF4E, allowing eIF4E to form the initiation complex and trigger translation [75,83,84]. After preincubation with the PI3K inhibitor, wortmannin, LPLI-induced phosphorylation of eIF4E and PHAS-I is abolished and LPLI-induced expression of cyclin D1 is reduced [76] (Fig. 1). Thus, these reports suggest that LPLI initiates protein translation and induces cell proliferation partly via the TPKR/PI3K/Akt/mTOR/eIF4E pathway.

#### PI3K/Akt/eNOS pathway

Nitric oxide (NO) has been shown to promote angiogenesis and vasculogenesis, which are the indispensable processes for tissue growth [85,86]. Endothelial NO synthase (eNOS) produces NO constitutively at low levels, which can be transiently stimulated to produce high levels of NO by hormones or various extracellular stimuli [87,88]. The proliferation and migration of endothelial cell play critical roles in angiogenesis [89]. Many growth factors, via PI3K/Akt/eNOS signaling pathway, have been reported to modulate endothelial cell proliferation, migration and angiogenesis [90-92]. He-Ne laser irradiation at 632.5 nm has been reported to increase human umbilical vein endothelial cell (HUVEC) proliferation, migration, NO secretion and promote angiogenesis [93]. During this process of LPLI stimulation, LPLI increases eNOS protein expression and gene expression (less than 0.26 J/cm^2^) in endothelial cells. The increased eNOS expression is inhibited by PI3K inhibitor LY294002, indicating that the activation of PI3K/Akt pathway is a critical step for the increased expression of eNOS upon LPLI [93] (Fig. 1). Thus, these findings suggest that PI3K/Akt/eNOS pathway is involved in the endothelial cell proliferation induced by low power He-Ne laser irradiation.

#### TPKR/PLC-gamma/PKC pathway

Activated TPKR also can activate the catalytic activity of phospholipase C (PLC)-gamma [28,94]. The activated PLC could catalyze the hydrolysis of some phospholipids, and then increase the concentration of diacylglycerol (DAG) and inositol triphosphate (IP3) in the cytoplasm. IP3 causes the endoplasmic reticulum (ER) to release Ca^2+ ^which works with DAG to activate PKCs [95-97]. By using the specific inhibitors, TPK/PLC/PKC/NADPH oxidase signal transduction pathways has been suggested to participate in the He-Ne laser-induced respiratory burst of neutrophils [28].

PKCs are involved in different functions, including the cellular proliferation, tumor promotion, differentiation, and apoptosis of many types of cells [95,98]. PKC was first described as a Ca^2+ ^activated, phospholipid-dependent serine/threonine protein kinase [99,100]. The 13 members of the PKC family can be divided into three subfamilies, based on their second messenger requirements: conventional (or classical) PKCs, requiring Ca^2+ ^and DAG for activation; novel PKCs, requiring DAG, but not requiring Ca^2+ ^for activation; and atypical PKCs, requiring neither Ca^2+ ^nor DAG for activation [101,102]. In our previous study, we investigated the involvement of PKCs in LPLI (He-Ne laser, 632.8 nm)-induced human lung adenocarcinoma cells (ASTC-a-1) proliferation [21]. By using laser scanning confocal microscope and fluorescence resonance energy transfer (FRET) technique, we showed the increased activation of PKCs in living cells during LPLI-induced proliferation. The increases of Ca^2+ ^concentration [24,28-30] and DAG in the cytoplasm have also been observed in the process of respiratory burst of neutrophils induced by LPLI [28]. Thus, we suppose that the Ca^2+^- and DAG-dependent classical PKCs may be involved in this process and that LPLI-induced cell proliferation is partly via the TPKR/PLC-gamma/PKC pathway (Fig. 1).

#### ΔΨm/ATP/cAMP/JNK/AP-1 pathway

Recent study demonstrated that He-Ne laser (632.8 nm) irradiation induces an immediate increase in mitochondrial membrane potential (ΔΨm), ATP, and cAMP of melanoma cell line A2058 cell via enhanced cytochrome c oxidase activity [39]. The ΔΨm is a sensitive indicator for the energetic state of the mitochondria and therefore the whole cells [103]. Later, LPLI promotes phosphorylation of Jun N-terminal kinase (JNK) and results in an activation of the transcription factor activator protein-1 (AP-1). The cell proliferation induced by LPLI is significantly abolished by the addition of chemicals that decreases ΔΨm or inhibits JNK. These results are not consistent with the above mentioned result that the activation of MAPK/ERK, but not JNK and p38 MAPK kinases, is involved in the proliferation of quiescent satellite cells upon LPLI [69]. It is probably that these two cells initiate different signaling pathways during proliferation induced by LPLI.

JNK is activated by growth factor stimulation and is phosphorylated primarily in response to cellular stress [104]. AP-1, an downstream kinase of JNK, promotes expression of genes involved in cell survival, proliferation, and angiogenesis [105]. cAMP, an important intracellular messenger, is capable of regulating differentiation, proliferation, and synaptic plasticity. Light irradiation causes cAMP elevation, which in turn stimulates both DNA and RNA synthesis [106]. cAMP analog significantly enhances the phosphorylation of JNK [39]. cAMP elevating agents can induce transcription factor AP-1 activity [107].

Taken together, these findings suggest that the signaling pathway cytochrome c oxidase/ΔΨm/ATP/cAMP/JNK/AP-1 is involved in the regulation of melanoma cell proliferation induced by LPLI. This investigation binds together light action upon the photoacceptor (putatively cytochrome c oxidase), modulation of elements of mitochondrial retrograde signaling (ΔΨm) and cell proliferation via AP-1[39].

#### ROS/Src pathway

LPLI could induce the production of reactive oxygen species (ROS) [9,12,22,24,34-37], which functions as the key secondary messengers regulating the activity of various protein kinases [40,108]. Non-receptor tyrosine kinases, particularly the Src kinases, are well-known targets of ROS and can be activated by oxidative events [109]. Beside the well known C-terminal tyrosine dephosphorylation activation system, Src is proposed to possess redox switch. It has been recently reported that Src is directly oxidized by the burst ROS during integrin-mediated cell adhesion and that this oxidation, which involves two sulfhydryl groups, namely, Cys245 in the SH2 domain and Cys487 in the kinase domain, leads to the enhanced activation of c-Src [110].

Src family kinases play key roles in regulating the fundamental cellular processes, including cell proliferation, attachment, migration and survival [111]. In our recent study, we investigated the involvement of Src in LPLI (He-Ne laser, 632.8 nm)-induced biostimulatory effects [22]. We firstly showed that LPLI increased the intracellular ROS level. Using a Src reporter based on fluorescence resonance energy transfer (FRET) technique and confocal laser scanning microscope, we visualized the dynamic Src activation in HeLa cells immediately after LPLI. Moreover, Src activation by LPLI was in a dose-dependent manner. In the presence of vitamin C, catalase alone, or the combination of catalase and superoxide dismutase (SOD), the activation of Src by LPLI was significantly abolished. Our results demonstrated that it was ROS that mediated Src activation by LPLI and that LPLI-induced change of cell viability is partly via the ROS/Src pathway. Taken together, our findings show the connection between the element of mitochondrial retrograde signaling (ROS) and secondary cellular reactions of Src activation, finally leading to the changes of HeLa cell viability.

#### The involvement of Bcl-2, BAX, p53 and p21 in LPLI-induced survival

The BCL-2 family members are major regulators of the apoptotic process. This family is comprised of pro-apoptotic (e.g. BAX) and anti-apoptotic (e.g. Bcl-2, Bcl-Xl) molecules [112]. Activated p53 mediates growth arrest and apoptosis by activating the expression of a number of cellular genes like p21 and BAX [113,114]. Under serum-deprivation conditions normally leading to apoptosis, LPLI (He-Ne laser, 632.8 nm) stimulation has been shown to promote the survival of fibers and their adjacent satellite cells, as well as cultured myogenic cells [20]. Upon LPLI stimulation, the expression of the anti-apoptotic protein Bcl-2 is markedly increased, whereas expression of the proapoptotic protein BAX is reduced. In the meanwhile, the expressions of p53 and the cyclin-dependent kinase inhibitor p21 are reduced [20]. The protective effect of elevating Bcl-2 expression in response to LPLI could be mediated by the suppression of p53 expression [114], or LPLI directly induces expression of Bcl-2 at the posttranscriptional level [20]. These findings imply that the protective effects of LPLI against apoptosis and promoting survival are partly mediated by regulation of these factors.

### Cell cycle-specific proteins involved in cell cycle progression induced by LPLI

**Figure 2:**
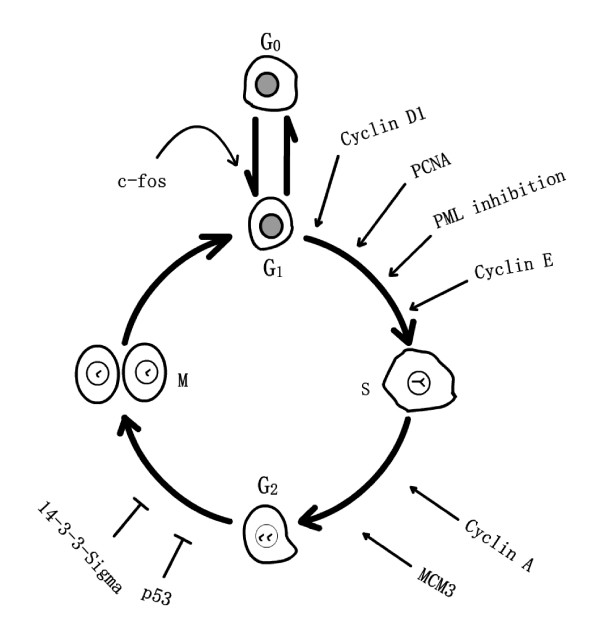
**Cell cycle-specific proteins involved in cell cycle progression induced by LPLI**.

The cell cycle is a crucial event in cell biology that consists of a series of repeated events allowing the cell to grow and duplicate correctly. Normal eukaryotic cell cycle consists of the following four discrete phases (Fig. 2): G1 (the time after cytokinesis and before the S phase), S (DNA synthesis), G2 (the time between S and M phase), and M (cell mitosis) [115,116]. By summarizing a large number of experiments the researchers proposed that the G1 phase of the mammalian cell could be subdivided into two phases, the early G1 phase and the later G1 phase. Cells in the early G1 phase could enter the G0 phase, and those in the later G1 phase could not [117]. G0 phase is the dormancy phase when the cell is not really doing anything at all. The point of discrimination is associated with the restriction point of cell cycle [118]. The normal progression of four phases of cell cycle is very important for normal cell growth.

#### c-fos

The *c-fos *proto-oncogene can be differentially regulated by various mitogenic agents at the transcriptional level during cell-cycle progression, differentiation, and development [119-121]. Ca^2+ ^can modulate the expression of a number of early response eukaryotic genes in isolated cells, however, *c-fos *gene expression merits special attention [120-122]. In recent study, He-Ne laser (632.8 nm) irradiation causes the increase of ΔΨm in isolated hepatocytes and the uptake of Ca^2+ ^by mitochondria, the latter could account for stimulation of energy metabolism [123]. Later, light irradiation triggers the activation of Ca^2+^-dependent *c-fos *gene and elevates the expression of *c-fos *in isolated hepatocytes [123]. These results show the connection between the elements of mitochondrial retrograde signaling (ΔΨm and Ca^2+^) and secondary cellular reactions of *c-fos *gene expression in response to LPLI. It has been reported that the expression of *c-fos *gives rise to a G0/G1 transition of the cell cycle in isolated hepatocytes [124]. Thus, it is possible that the elevated expression of *c-fos *induced by LPLI promotes the quiescent cell leave G0 phase and enter G1 phase (Fig. 2).

#### Cyclin D1, cyclin E, cyclin A and PCNA

Cyclin D1 is a critical target for proliferative signals and required for cell-cycle progression in G1 phase [125]. Cyclin E is induced at the G1/S boundary, and cyclin A is induced at a later phase of the cell cycle and is required for the cell to progress through the S phase [126]. Three seconds of He-Ne laser (632.8 nm) irradiation increases the expressions of cell-cycle regulatory proteins: cyclin D1, cyclin E and cyclin A in mouse satellite cells, pmi28. The induction of cyclin D1 expression is detected as early as 6 minutes after irradiation, suggesting that LPLI increases the protein synthesis and that LPLI initiates the translation of proteins that are required for entrance and progression through the G1 phase of the cell cycle [76]. It has been reported that sustained activation of ERK1 is required for the continued expression of cyclin D1 in the G1 phase [127,128] and that the sustained activation of ERK1 is detected during LPLI-induced cell proliferation [69]. LPLI stimulation also upregulates the expression of another early cell-cycle protein, proliferating cell nuclear antigen (PCNA), in the late G1 phase in primary rat satellite cells [19]. These findings imply that laser irradiation allows the cells to pass through G1 phase and enter S phase by affecting early cell-cycle regulatory genes, and that cell proliferation increases at last (Fig. 2).

#### PML

Checkpoint regulator proteins control entrance of the cell cycle from a quiescent state, as well as progression in the cell cycle. One such protein, the promyelocytic leukemia protein (PML), blocks the entry of the cells to S phase of the cell cycle [129]. PML protein is typically concentrated in subnuclear PML oncogenic domains (PODs) [20], together with other important cell cycle regulatory proteins like p53. The localization and distribution of PML in the cells are related to the cell cycle progression [21]. The redistribution of PML from PODs to the nucleoplasm is considered to be the evidence that the cell is leaving the quiescent stage and entering the S phase[31].

Upon LPLI of human keratinocytes with light at 780 nm, the ΔΨm is increased immediately and the subnuclear distribution of PML is altered from discrete domains to its dispersed form within less than 1 hour. By 3 hours after irradiation, the intensity of PML fluorescence is markedly reduced compared with control cells, suggesting that the PML protein is largely degraded [31]. The ΔΨm is a sensitive indicator for the energetic state of the mitochondria and therefore the whole cells [103]. These results show the connection between the element of mitochondrial retrograde signaling (ΔΨm) and secondary cellular reactions of the alteration of PML distribution in response to LPLI. Thus, LPLI may induce cell cycle progression from G1 to the S phase through redistribution and degradation of PML protein, thereby enabling cell proliferation [31] (Fig. 2).

#### MCM3

Minichromosome maintenance deficient 3 (MCM3) as licensing factor is involved in the initiation of DNA replication in eukaryotic cells. Upon LPLI (gallium-aluminum-arsenide (Ga-Al-As) diode laser), mRNA levels of MCM3 are elevated in laser-irradiated osteoblast cells compared with the levels in control cells. Furthermore, radiolabelled thymidine incorporation is increased under laser-irradiation conditions. These findings imply that LPLI enhances DNA replication mediated by the enhancement of the MCM3 gene expression and plays an important role in proliferation of osteoblast [130] (Fig. 2).

#### 14-3-3-sigma and phospho-p53

LPLI (gallium-aluminum-arsenide (Ga-Al-As) diode laser) induces differentiation of osteoblastic cells by the increases of Runx2 expression and ALP-positive colonies. By 12 hours after irradiation, a higher proportion of cells are in the G2/M boundary of the cell cycle compared with the control. This result is confirmed by the appearance of G2/M arrest marker 14-3-3-sigma or phospho-p53. These findings demonstrate that LPLI induces not only acceleration of bone formation but also initiation of G2/M arrest, which may promote wound healing [131] (Fig. 2).

### Signal molecules involved in inflammatory response reduced by LPLI and immunomodulatory effects enhanced by LPLI in animal modes

#### Signal molecules involved in inflammatory response reduced by LPLI in animal modes

Transcription factor nuclear factor kappa B (NF-κB) belongs to the Rel family of DNA-binding proteins and is present in the cytoplasm in an inactive state as a dimer bound with the inhibitory IκB protein [132]. A variety of external stimuli, such as ROS, proinflammatory cytokines, or phorbol esters, can activate NF-κB via phosphorylation of IκB by IκB kinases (IKK) [132]. Activated NF-κB can enhance the expression of different genes, and is essential for the activation of inducible form of nitric oxide synthase (iNOS) expression in rat gastrocnemius muscle and skeletal muscle myocites [133-135].

During the inflammatory phase of trauma, factors like phagocytic stimuli increase the production of reactive oxygen species (ROS), resulting in oxidative stress [136,137]. It has reported that ROS may directly damage vital cell constituents, such as lipids, proteins, and DNA [138], and that ROS also can activate NF-κB that is accompanied with increased degradation of its inhibitor IκB. The activated NF-κB, in turn, increases the expression of the iNOS and subsequent synthesis of NO [133,135]. NO and its reactive nitrogen intermediates may destruct cells and tissues and play a significant role in the pathology of many inflammatory conditions [139].

In an animal mode, upon LPLI (galium arsenide (Ga-As) laser, 904 nm), the release of ROS induced by trauma is blocked and the activation of NF-κB induced by trauma is also blocked. In the meanwhile, the reduction of the trauma-induced IκB protein is significantly inhibited. Furthermore, LPLI also reduces the associated overexpression of iNOS and production of collagen. These findings suggest that LPLI reduces the inflammatory response induced by trauma in the gastrocnemius muscle of rat [140].

In the lung neutrophils obtained from mice subjected to lipopolysaccharide (LPS)-induced lung inflammation, the mRNA levels of anti-apoptotic Bcl-xL and A1 mRNA are increased. When the mice are treated with LPLI (660 nm diode laser) and an inhibitor of NF-κB nuclear translocation (BMS 205820), the mRNA levels of Bcl-xL and A1 mRNA are decreased in lung neutrophils. Thus, LPLI reduces the levels of anti-apoptotic factors by an action mechanism in which NF-κB seems to be involved [141]. These findings indicate that LPLI reduces the inflammatory response induced by LPS in LPS inflamed mice lung neutrophils. The decreased mRNA level of anti-apoptotic protein Bcl-xL after LPLI in this animal mode is not consistent with the result that LPLI increases the expression of anti-apoptotic protein Bcl-2 in cultured cells [20]. Thus, it is impossible that LPLI-reduced inflammatory response and LPLI-induced cell survival are through the exact same mechanisms. We suppose that the human (or animal) body not single cell keeps the balance between normal developments induced by promotive elements and abnormal developments induced by pathological elements. The abnormality predominates in the body developments under pathological conditions. LPLI stimulation serves as a very advantageous regulating element by inhibiting the abnormal developments and strengthening immunity against pathological conditions.

#### Signal molecules involved in immunomodulatory effectsenhanced by LPLI in animal modes

The increased immunomodulatory effect is observed in the following animal model. Using periodic He-Ne laser radiation on the thymus zone of animal for 1 month, the authors detect more pronounced changes in the cytokine production as well as in NO and Hsp70 synthesis. Thus, LPLI shows more effective immunomodulatory effects when applied on the thymus projection area of animal [142].

### Expression and secretion of molecules induced by LPLI

#### Expression and secretion of growth factors induced by LPLI

It has been shown that LPLI significantly increases the gene or protein expressions of several growth factors (Table 1), including brain derived neurotrophic factor (BDNF) and glial derived neurotrophic factor (GDNF) in olfactory ensheathing cells [143], bFGF growth factor production in fibroblasts [144], insulin-like growth factor (IGF)-I in the calvarial cells [145], keratinocyte growth factor (KGF) in human keratinocytes [31], platelet-derived growth factor (PDGF) in cultured fibroblasts[146], transforming growth factor-β (TGF-β) in the cardiomyocytes [18,147], transforming growth factor-β1 (TGF-β1) [39,148,149], vascular endothelial growth factors (VEGF) by smooth muscle cells, fibroblasts, cardiomyocytes and cardiac myocytes [18,147,150]. Among these growth factors, before up-regulation of KGF in keratinocytes [31] and release of TGF-β1 from melanoma cells [39], the researchers examine the immediate increase in ΔΨm level after light irradiation, which suggests the connection between the elements of mitochondrial retrograde signaling and secondary cellular reactions of expression and secretion of growth factors. Accompanied with increased expressions of these growth factors, the cells exhibit the corresponding biological effects induced by LPLI, such as cell proliferation, differentiation and bone nodule formation.

**Table 1:** Modulation of expression and secretion of molecules by LPLI

Classification	Molecules	Biological effects of LPLI
Growth factors	BDNF, GDNF, bFGF, IGF-I, KGF, PDGF, TGF-β, VEGF	Proliferation, Differentiation, Bone nodule formation
		
Interleukins	IL-1α, IL-6, IL-8, IL-2, IL-4	Proliferation, Migration, Immunological activation
		
Inflammatory cytokines	PGE2, COX-2, IL-1β, TNF-α	Inhibition of inflammation
		
Small molecules	ATP, cGMP, ROS, Ca^2+^, NO	Normalization of cell function, Pain relief, Healing, Mediating cell activities, Migration, Angiogenesis

#### Expression and secretion of interleukins induced by LPLI

Following irradiation of human keratinocytes with light at 780 nm, the ΔΨm is increased immediately and then the expression of interleukin-1alpha (IL-1α) and interleukin-6 (IL-6) are transiently unregulated (Table 1) [31]. He-Ne irradiation of melanoma cell line A2058 cells induces an immediate increase in ΔΨm, ATP, and cAMP via enhanced cytochrome c oxidase activity and results in delayed effects on interleukin-8 (IL-8) release [39]. IL-1α, IL-6 and IL-8 could provoke proliferation of keratinocytes and melanoma cells, respectively; moreover, IL-1α also could induce keratinocytes migration. It is possible that these cytokines play a critical role in the enhancement of keratinocyte and melanoma cells proliferation and migration of keratinocyte induced by LPLI. These results show the connection among photoacceptor (cytochrome c oxidase), element of mitochondrial retrograde signaling (ΔΨm), secondary cellular responses of expression and secretion of IL-1α, IL-6 and IL-8, and cells proliferation and migration. In addition, the levels of IL-2 mRNA and IL-4 mRNA after irradiation of skin are increased, which suggests that LPLI affects the cutaneous immunological activation [151].

#### Expression and secretion of inflammatory cytokines induced by LPLI

After LPLI, the decreased expressions of several inflammatory cytokines are observed (Table 1). Lipopolysaccharide (LPS) is known as a potent stimulator of inflammation [152]. In human gingival fibroblast (hGF) cells, LPLI (Ga-Al-As diode laser) significantly inhibits production of prostaglandin E2 (PGE2) and decreases the mRNA levels of cyclooxygenase-2 (COX-2) induced by LPS [152]. In human keratinocytes and porcine aortic SMC, after irradiation, the expression of the proinflammatory gene interleukin-1beta (IL-1β) is suppressed, this finding reflects the suppression of inflammation by LPLI [15,31]. Before the suppression of IL-1β expression in keratinocytes, the increase of ΔΨm is monitored immediately after LPLI, which suggests the connection between element of mitochondrial retrograde signaling (ΔΨm) and secondary cellular responses. In Wistar rats, LPLI (650-nm Ga-Al-As laser) stimulation reduces expression of TNF-α, a potent pro-inflammatory cytokine, after acute immunocomplex lung injury [153].

#### Increased release of some small molecules induced by LPLI

In addition to the molecules mentioned above, LPLI also increases the production of some small molecules (Table 1). LPLI could induce the production of ATP, which leads to normalization of cell function, pain relief, and wound healing. The sufficient cellular energy enables cells to work normally and is essential for successful self-healing process [55,154-159]. LPLI could induce the production of cyclic guanosine monophosphate (cGMP) in human corpus cavernosum smooth muscle cells (HCC SMC) [160]. LPLI also could induce the production of ROS [9,12,22,24,34-37] and elevation of intracellular Ca^2+ ^concentration [24-30]. The generation of small amounts of ROS, as natural signal messengers, has been considered to be important for regulating cell activities [22,35-37]. Each transient Ca^2+ ^signal is characterized by an amplitude and a duration, which are specific in their signaling events [161]. This signal can be interpreted by the cells. For example, the elevation of intracellular Ca^2+ ^concentration or the complex frequency of Ca^2+ ^oscillations are correlated with the spatio-temporal activation of Ras protein through the ERK/MAPK cascade [162]. In addition, LPLI can induce the production of NO in human umbilical vein endothelial cell (HUVEC) [93], which promotes the cell migration and angiogenesis [85,86,163,164].

### The reasons why LPLI has different biologic effects on different cells

It appears that LPLI has different biologic effects on different cells, and the followings are our comments on this issue. First, we analyze these differences from the capabilities of cell responding to LPLI. One key secondary reaction invoked by LPLI is electronic excitation of the mitochondrial respiratory chain components [45], which leads to the production of ATP and various metabolic processes, such as the synthesis of DNA, RNA, proteins, enzymes, and other products needed to repair or regenerate cell components [12]. The specificity of final photobiological response is determined not at the level of primary reactions in the respiratory chain but based on secondary cellular signaling cascades [44]. In pathological cells, these secondary cellular responses are inhibited [165]. Thus, LPLI will have much more pronounced effects due to the reduced cellular responses [166]. If the cellular environment is optimal or near optimal (nonpathological), the effects of LPLI will not be as pronounced [166]. In essence, LPLI acts to normalize cellular function, which is important to understand some of the varying results of LPLI experimentation in the literatures [166]. Secondly, we analyze the different biological effects of LPLI from the redox status of the cells. It has been shown that the biological effects of LPLI are dependent on the initial redox status of the irradiated cells. The mitochondrial retrograde signaling pathways have been suggested to be influenced by intracellular redox balance [23]. In many cases, the regulative role of redox balance has proved to be more important than that of ATP [23]. The cells whose overall redox potential is shifted to a more reduced state (e.g., certain pathological conditions) are more sensitive to irradiation. In contrast, cellular response is weak or absent when the redox potential of the irradiated cell is optimal or near optimal. This explains why LPLI has different biologic effects on different cells and why the biologic effects are sometimes nonexistent [44].

The above two explanations are analyzed from the different initiate status of the irradiated cells, but the light-induced biological effects also depend on the parameters of the irradiation (wavelength, dose, intensity, radiation time, continuous-wave or pulsed mode, pulse parameters) [9,32,44,51,55]. For example, Karu [23] mentioned that red light at 650 mn increased oxidative phosphorylation, but far-red light at 725 nm inhibited it when irradiating isolated rat liver mitochondria, and that irradiation at 670 and 830 nm stimulated the proliferation of the Schwann cells, but irradiation at 780 nm inhibited it. It is probably that the wavelengths mentioned above are absorbed by different chromophores in a different redox state and that different absorbing chromophores may play a different role in driving the metabolism [23]. Another example, our recent cellular viability assay revealed that laser irradiation of low doses (≤ 25 J/cm2) promoted HeLa cells viability while high doses impaired [22]. This indicates that some beneficial effects may be lost if LPLI doses are too high.

Due to the individual irradiated cell or individual parameter of the irradiation in different literatures, different cells exhibit different biologic effects. According to the cDNA microarray technique, the different biologic effects after LPLI can be grouped into several categories. The gene expression profiles of human fibroblasts reveals that 111 genes of 10 function categories are upregulated by the low-intensity red light irradiation [167]. Among these 10 function categories, seven are directly or indirectly involved in the enhancement of cell proliferation. The other three function categories upregulated are genes related to transcription factors, immune/inflammation and cytokines as well as some genes not identified [42,167]. Thus, these biological effects upon LPLI including promotion of proliferation, regulation of immune/inflammation, expression of transcription factors and cytokines agree with our above reviewed photobiological effects. Please note that the cells derived from different classifications will be characterized as the specific responses upon LPLI. For example, endothelial cells involved in many aspects of vascular biology (such as vasoconstriction, vasodilation and angiogenesis) exhibit increased proliferation and migration after LPLI [93]. The proliferation and migration of endothelial cell play critical roles in angiogenesis [89]. Mesenchymal stem cells (MSCs) and cardiac stem cells (CSCs) [13], significant for implantation in regenerative medicine, show increased proliferation. Cell proliferation is a common response to LPLI. The cells coming from hematological system show their own features upon LPLI, such as erythrocytes with the improved deformability after laser irradiation [168]. In inflamed mice lung neutrophils induced by lipopolysaccharide (LPS), LPLI stimulation reduces the inflammatory response [141]. Helium-neon laser radiation (632.8 nm) on the thymus projection area of mice causes more effective immunomodulatory effects in T cells [142].

### Perspectives

Clinical applications for low power laser therapy (LPLT) include wound healing, pain attenuation and various forms of inflammation [33,169]. At present, there are a large number of clinical trials using low power laser therapy, but there are a relative small number of basic researches of LPLI. Currently, the wavelengths, dosage schedules, and appropriate conditions of laser irradiation are not well established. Thus, to facilitate the physician to match optimally the laser in clinical practices, the burning issue is to study the basic mechanisms of the biological effects of LPLI. Recently, the researchers pay close attention to the signaling pathways involved in the biological effects of LPLI. It is believed now that extracellular stimuli trigger cellular responses such as proliferation, differentiation, and even apoptosis through the pathways of cellular signaling. Thus, we suppose that the investigation of the molecular events induced by LPLI could eventually reveal the mechanisms of LPLI. It is obvious that there are close connections between mitochondrial retrograde signaling and cellular molecular events such as the activation or suppression of kinases in the cytoplasm and subsequent changes of downstream cascades. The elements of mitochondrial retrograde signaling (ΔΨm, ROS, Ca^2+^, NO^•^, pH_i_, fission-fusion homeostasis of mitochondria) can mediate the cellular molecular events, but at present we study less about it. Further studies investigating the connections between mitochondrial retrograde signaling and cellular molecular events are needed for understanding the basic mechanisms of LPLI.

In this review, we summarized the studies on the molecular mechanisms of LPLI-induced proliferation since January 1999. Among these studies, one of the most prominent features is that advanced techniques extensively used in the study of cell biology are less used in the basic research of LPLI. Most results of the basic researches are obtained from traditional techniques in cell biology. Thus, to better reveal the molecular mechanisms of LPLI, we need combine the traditional techniques with the innovative techniques. The basic researches of LPLI will eventually guide the application of LPLI in clinical practices.

## Abbreviations

ΔΨm: mitochondrial membrane potential; AP-1: activator protein-1; ATP: adenosine triphosphate; BDNF: brain derived neurotrophic factor; cGMP: cyclic guanosine monophosphate; COX-2: cyclooxygenase-2; DAG: diacylglycerol; EGF: epidermal growth factor; eIF4E: Eukaryotic initiation factor 4E; eNOS: Endothelial NO synthase; ERK: extracellular signal-regulated protein kinase; FAD: flavin adenine dinucleotide; FMN: flavin mononucleotide; FRET: fluorescence resonance energy transfer; GDNF: glial derived neurotrophic factor; IGF: insulin-like growth factor; IL: interleukin-1alpha; iNOS: inducible form of nitric oxide synthase; IP3: inositol triphosphate; JNKs: Jun N-terminal kinases; KGF: keratinocyte growth factor; LPLI: low power laser irradiation; LPS: lipopolysaccharide; MCM3: Minichromosome maintenance deficient 3; mTOR: mammalian target of rapamycin; NF-κB: nuclear factor kappa B; NO: Nitric oxide; PCNA: proliferating cell nuclear antigen; PDGF: platelet-derived growth factor; PGE2: prostaglandin E2; PHAS-I: protein heat and acid stable: also as eIF4E binding protein-1 4EBP1; PI3Ks: Phosphoinositide 3-kinases; PLC: phospholipase C; PML: promyelocytic leukemia protein; PODs: PML oncogenic domains; PpIX: protoporphyrin IX; ROS: reactive oxygen species; SDH: succinate dehydrogenase; TGF-β: transforming growth factor-β; TNF-α: tumor necrosis factor α; TPKR: tyrosine protein kinase receptors; VEGF: vascular endothelial growth factor.

## Competing interests

The authors declare that they have no competing interests.

## Authors' contributions

XG drafted the manuscript. DX participated in the design and discussion of the study. All authors read and approved the final manuscript.
